# Using qPCR and microscopy to assess the impact of harvesting and weather conditions on the relationship between *Alternaria alternata* and *Alternaria* spp. spores in rural and urban atmospheres

**DOI:** 10.1007/s00484-023-02480-w

**Published:** 2023-05-16

**Authors:** Godfrey Philliam Apangu, Carl Alexander Frisk, Beverley Adams-Groom, Geoffrey M. Petch, Mary Hanson, Carsten Ambelas Skjøth

**Affiliations:** 1 School of Science and the Environment, University of Worcester, Henwick Grove, WR2 6AJ Worcester, UK; 2 Present Address: Protecting Crops and the Environment, Rothamsted Research, West Common, Harpenden, Hertfordshire AL5 2JQ UK; 3 Present Address: Department of Urban Greening and Vegetation Ecology, Norwegian Institute of Bioeconomy Research, Ås, Norway; 4 Present Address: Edith Cowan University, 270 Joondalup Drive, Joondalup, WA 6027 Australia; 5 Present Address: Department of Environmental Science, Aarhus University, Frederiksborgvej 399, 4000 Roskilde, Denmark

**Keywords:** Concentration, Fungi, Allergens, eDNA, Microbiology

## Abstract

*Alternaria* is a plant pathogen and human allergen. *Alternaria alternata* is one of the most abundant fungal spores in the air. The purpose of this study was to examine whether *Alternaria* spp*.* spore concentrations can be used to predict the abundance and spatio-temporal pattern of *A*. *alternata* spores in the air. This was investigated by testing the hypothesis that *A*. *alternata* dominates airborne *Alternaria* spp. spores and varies spatio-temporally. Secondarily, we aimed at investigating the relationship between airborne *Alternaria* spp. spores and the DNA profile of *A*. *alternata* spores between two proximate (~ 7 km apart) sites. These were examined by sampling *Alternaria* spp. spores using Burkard 7-day and cyclone samplers for the period 2016–2018 at Worcester and Lakeside campuses of the University of Worcester, UK. Daily *Alternaria* spp. spores from the Burkard traps were identified using optical microscopy whilst *A*. *alternata* from the cyclone samples was detected and quantified using quantitative polymerase chain reaction (qPCR). The results showed that either *A*. *alternata* or other *Alternaria* species spores dominate the airborne *Alternaria* spore concentrations, generally depending on weather conditions. Furthermore, although *Alternaria* spp. spore concentrations were similar for the two proximate sites, *A*. *alternata* spore concentrations significantly varied for those sites and it is highly likely that the airborne samples contained large amounts of small fragments of *A*. *alternata*. Overall, the study shows that there is a higher abundance of airborne *Alternaria* allergen than reported by aerobiological networks and the majority is likely to be from spore and hyphal fragments.

**Supplementary Information:**

The online version contains supplementary material available at 10.1007/s00484-023-02480-w.

## Introduction

*Alternaria* is a fungus, which is a plant and animal pathogen and human aeroallergen (Nowicki et al. 2012; Meena et al. 2017; Grinn-Gofroń et al. 2019). Human exposure to *Alternaria* spores causes allergy and severe asthma hospital admissions in sensitised individuals, more frequently in children (Neukirch et al. 1999; Mitakakis et al. 2001). Traditionally, optical microscopy is the main instrument for monitoring aeroallergens such as *Alternaria* (West et al. 2017). However, the use of morphological features alone to identify *Alternaria* spores at species level is unreliable due to their overlapping characteristics (Ammour et al. 2020; West et al. 2017).

*Alternaria alternata* is among the most prevalent fungal species causing sensitisation to allergy (Mari et al. 2003)*.* It is one of the abundant, allergenic and pathogenic *Alternaria* species among fungal types whose spores are frequently observed in the air (Gabriel et al. 2015, 2016). However, no study has examined the abundance of *A*. *alternata* within the *Alternaria* genus. *A*. *alternata* allergen (Alt a 1) is the most prevalent among patients sensitised to fungal allergy (López Couso et al. 2021). *A*. *alternata* has a high spatio-temporal variability due to changing weather, diverse host ranges and abundance of inoculum, making its occurrence, until now, generally unpredictable (Ding et al. 2021). It is unknown whether *A*. *alternata* spore concentrations differ between nearby rural and urban areas especially during periods of high spore concentrations. However, the metabarcoding approach used to analyse airborne microorganisms revealed systematic differences between the rural and urban atmosphere and high fungal spore biodiversity (Hanson et al. 2022). Spores of different *Fusarium* species were concurrently dispersed when meteorological variables simultaneously influenced their development and dispersion (Audenaert et al. 2009; Xu et al. 2005). Simultaneous growth and eventual atmospheric release of different *Alternaria* species within an area is very likely due to some species being host specific, whilst others may be opportunistic and associated with many hosts (Al-Lami et al. 2019). However, such a simultaneous relationship between *A*. *alternata* and *Alternaria* spp. spores has not been tested before especially for nearby rural and urban areas. Simultaneous dispersal of the spores of different *Alternaria* species increases exposure and severity of the symptoms of allergy and asthma in sensitised individuals (Damialis and Gioulekas 2006; Madsen et al. 2016). Meanwhile, the asynchronous spore dispersal pattern among the species, as observed between *A*. *alternata* and *A*. *solani*, prolongs disease virulence and severity in crops as well as the season of allergy (Al-Lami et al. 2020; Ding et al. 2021). Generally, *Alternaria* spp. spore concentrations have been shown to have a high spatio-temporal variation, due to the effect of the meteorological variables and anthropogenic activities, e.g. crop combine-harvesting that govern their sporulation and dispersal (Escuredo et al. 2011; Recio et al. 2012; Skjøth et al. 2012). However, it is unknown how the meteorological variables and combine-harvesting of crops impact *A*. *alternata* spore concentrations, especially at nearby rural and urban sites that are < 10 km apart.

Therefore, the purpose of the study was to understand whether the overall *Alternaria* spp*.* spore concentrations (obtained with optical microscopy) can be used to predict the distribution and spatio-temporal pattern of *A*. *alternata* spores in an area. This was achieved by examining the relationship between the DNA profile of *A*. *alternata* spores and overall *Alternaria* spp. spore concentrations between two proximate (~ 7 km apart) sites. We tested the hypothesis that airborne *Alternaria* spore concentrations are dominated by *A*. *alternata* and have varying spatio-temporal patterns. This was examined by detecting and quantifying *Alternaria* spp. spore concentrations using optical microscopy and *A*. *alternata* spore DNA profile using qPCR, at two separate nearby locations in one region. The impact of the meteorological variables and crop combine-harvesting on the spatio-temporal variations of the spores was examined.

## Materials and methods

### Spore sampling instruments and locations

*Alternaria* spp. spores were sampled using pairs of Hirst-type Burkard 7-day volumetric spore trap (Hirst 1952) and an automatic multi-vial cyclone sampler (Burkard Manufacturing, UK) at the University of Worcester, Worcester, UK. The spores were sampled (Fig. S1) at a rural (Lakeside campus) and an urban site (Worcester campus) continuously for approximately 3 months (Jul–Sep) for three separate years (2016–2018; Table S1) during the main *Alternaria* spore season in the areas (Apangu et al. 2020). Worcester is an urban area surrounded by agricultural areas comprising permanent orchards for fruit and cider production (Sadyś et al. 2014), crops under rotation (Apangu et al. 2020; Sadyś et al. 2015), grasslands and pasture within the public parks (Sadyś et al. 2016) and small woodlands (Skjøth et al. 2015). The Lakeside site is a rural area whose immediate vicinity had no source of *Alternaria* spores (e.g. buildings in the south, non-vegetated areas such as hard standing, roadways and a man-made lake in the west). However, areas outside the vicinity comprised potential sources such as pine trees, grassland, mixed arable crop fields, permanent pastures, animal paddocks and patches of trees. Lakeside samplers were approximately 7 km away from Worcester.

Sampling heights were set following the recommendations on the height of pollen/spore sampling (Galán et al. 2014). Samplers at Worcester (52.1970 N, − 2.2421 E) were placed 10 m above ground level (AGL) on the rooftop of the Edward Elgar building (e.g. Sadyś et al. 2014) to capture spores at both local and regional scales. Meanwhile, samplers at Lakeside (52.2544 N, − 2.2537 E) were placed on top of a container (hereafter Lakeside Container) 4 m AGL. Sampling *Alternaria* spores using the Burkard trap was conducted similarly to the previous studies (Adams-Groom et al. 2020; Apangu et al. 2020). Meanwhile, the particles collected by the cyclone sampler were deposited by centrifugal force through spiralling action into a 1.5 mL microcentrifuge tube, similar to Pashley et al. (2012). The cyclones automatically replace the microcentrifuge tube with a new one at 9:00 am to ensure that daily samples from the cyclones are temporally synchronised across the sampling network (Brennan et al. 2019). The samples of the 7-day Burkard trap at Worcester were unloaded weekly at 9:00 am whilst those at Lakeside Container were emptied at 14:00 and spore data was later converted to match that at Worcester. The Burkard trap samples were stored at 4 °C until they were mounted on microscope slides. The microscope slides were prepared and *Alternaria* spores were identified and counted according to a standard procedure used for over 50 years in England and other European countries (Adams-Groom et al. 2020; Apangu et al. 2020; BAF 1995; Kasprzyk 2008; Makra et al. 2010; Skjøth et al. 2016). Samples collected with the cyclone sampler were stored at – 20 °C for about a month and then transferred to – 80 °C for long-term storage in order to preserve the spores until the DNA extraction. This procedure limits DNA degradation over time, a common problem with environmental samples (Lear et al. 2018). Here, storage at – 80 °C for a year (Pashley et al. 2012) or several years (Hanson et al. 2022) has successfully been applied to air samples collected with cyclones.

### Microscopic *Alternaria* spp. spore identification

*Alternaria* spp. spores from the Burkard trap were identified using an optical microscope (Sadyś et al. 2014) and counted using the 12 transverse method at × 400 magnification according to fungal spore monitoring recommendations in Europe (Galán et al. 2021). This approach is being used for fungal spore monitoring in Worcester, UK (Apangu et al. 2020), Denmark (Skjøth et al. 2012) and Hungary (Paldy et al. 2014). The daily (24 h) mean *Alternaria* spp. spore concentrations were expressed as spores m^−3^ of air by multiplying the microscopic spore counts with previously calculated correction factors (Lacey and Allit 1995). To match the 7-day *A*. *alternata* spore data (spores, hyphal and mycelial fragments) from the cyclone samplers, weekly mean *Alternaria* spp. spore concentrations were calculated by summing *Alternaria* spp. spore concentrations every 7 days and computing their averages.

### Cultivation and quantification of *A. alternata* conidia as reference for qPCR

*A*. *alternata* conidia were cultivated and later used in qPCR bioassays to identify and quantify airborne *A*. *alternata* spores sampled at Lakeside Container and Worcester. To achieve this, conidia of *A*. *alternata* (Culti-Loops™ TX 8025) procured from Fisher Scientific, UK, were grown under sterile conditions on potato dextrose agar for 23 days at 23 °C, similar to Smith et al. (2012). The Culti-Loops™ *A*. *alternata* isolate was originally derived from the American Type Culture Collection (ATCC®). To harvest spores, sterile water (10 mL) was added to the culture Petri dish and spore suspensions were obtained by gently scraping the surface of the culture using a sterile L-shaped spreader. Five millilitres of the spore suspension was drawn into a clean and sterile 50 mL microcentrifuge tube. The spore suspension was recovered after 5 min of centrifugation (CL31 Multispeed centrifuge, Thermo Scientific) at 2500 rpm, corresponding to 600 × *g.* The supernatant was discarded and the pellet was transferred into a clean 2 mL microcentrifuge tube and resuspended in 1 mL sterile water. Ten microliters of the spores from the pellet suspension were counted using an improved Neubauer haemocytometer (Nexcelom Bioscience 2018), similar to Ojaghian et al. (2018), where the Neubauer haemocytometer is one of several types of counting chambers designed for counting cells in a liquid. In this case, the counting chamber has a depth of 0.1 mm. The concentration of *A*. *alternata* spores per mL was calculated using the formula below (Nexcelom Bioscience 2018). If the spore concentration is low as in our case, then it is recommended to count all the 9 large squares in the haemocytometer which we did here, whilst the alternative is to count only the central square. The pellet suspension with a known spore concentration was then used for generating standard curves (‘real-time qPCR’ section) for qPCR and used for computing mean spore concentrations in the air samples.

$$\mathrm{Spore\;concentration\;}\left(\mathrm{Spores\;}{\mathrm{mL}}^{-1}\right) = \frac{\mathrm{Total\;spore\;count}}{\mathrm{No}.\mathrm{\;of\;}1{\mathrm{\;mm}}^{2}\mathrm{\;squares\;counted}} \times {10}^{4}$$

In our case, the spore count in each of the nine grids was 201, 112, 209, 164, 185, 151, 174, 160 and 209, respectively. This corresponds to a mean spore concentration in the pellet suspension of 1.74*10^6^ spore mL^−1^.

### DNA extraction from air samples and culture material

DNA was isolated from the daily air samples collected with multi-vial cyclone samplers and the *A*. *alternata* culture material using a commercial protocol (Fast DNA spin kit for soil; MP Biomedicals), similar to previous studies (Chen et al. 2020; Degois et al. 2019; Ettenauer et al. 2012; Fröhlich-Nowoisky et al. 2012). Cell lysis solution (CLS)-Y buffer, which aids release of impacted DNA in the spores through enzymic cell membrane breakdown, was added to the daily air samples (100 μL) and culture material (200 μL). The daily air samples and culture material were then vortexed using the Vortex-Genie 2 vortex mixer (Mo Bio Laboratories, Inc) at a maximum speed (ten) and for 5 min. The daily air samples were pooled every seven consecutive days of spore sampling to form a 7-day pooled air sample of 700 μL, similar to Brennan et al. (2019). The 7-day pooled air samples and culture material were diluted to 1 mL by topping up with 300 and 800 μL of CLS-Y buffer, respectively and samples transferred into a 2 mL lysing matrix A tube (pre-filled with irregularly shaped garnet particles and a single 1/4 inch ceramic sphere). The 7-day pooled air samples and culture material were then homogenised using a FastPrep® instrument (MP Biomedicals) for 40 s at 6 m/s. The rest of the steps in the Fast DNA spin kit protocol were followed for the DNA extraction from the 7-day pooled air samples and culture material. DNA was eluted by resuspending the binding matrix above the SPIN filter in 100 μL of DES (DNase/Pyrogen-free water). The concentration of DNA in the samples was quantified using a Nanodrop 2000c spectrophotometer instrument (Fisher Scientific, UK), similar to previous studies (Degois et al. 2019; Ettenauer et al. 2012; Hanson et al. 2022; Shokere et al. 2009). The DNA was stored at – 20 °C for subsequent analyses.

### Real-time qPCR

To detect and quantify the amount of DNA from *A*. *alternata* spores in the air samples, qPCR assays were conducted according to experimental conditions stipulated in Black et al. (2013). For each qPCR assay, a 25 μL reaction volume was prepared to contain 2 μL template DNA from air samples, 12.5 μL qPCRbio SyGreen Blue mix (PCR Biosystems, UK), 1.2 μM of each forward and reverse primer and 4.5 μL purified water (Sigma, UK; Black et al. 2013). *A*. *alternata* was detected using the primers; Sense 5′ CGA ATC TTT GAA CGC ACA TTG 3′, Antisense 5′ CGC TCC GAA ACC AGT AGG 3′ (Black et al. 2013). To check for the primer specificity to *A*. *alternata*, melt curve temperature was analysed and the primer sequences were BLAST searched in the NCBI Genbank database, similar to Patel et al. (2019). DNase-free double-distilled H_2_O (Clent Life Science, UK) replaced DNA in the master mix as a negative control. The qPCR amplification was conducted in a Roche LightCycler 480 and analysed using Gene Scanning software machine v.1.5 (Roche Molecular Sytems, UK) with each sample and standard prepared from culture extract run in triplicate. DNA in the samples was initially denatured at 95 °C for 4 min. Sample products were detected and amplified at 95 °C for 40 cycles and 10 s, followed by 57 °C for 10 s and 72 °C for 10 s. Melting curve parameters were set at 95 °C for 1 min, followed by 40 °C for 1 min, 60 °C for 1 s and 95 °C with a continuous analysis mode. The products were cooled at 40 °C for 30 s. Fluorescence levels were recorded at the end of each amplification cycle as stipulated in the minimum information for publication of quantitative real-time experiments guideline (Bustin et al. 2009). The qPCR method only works by having a standard curve based on a set (here 7) of known concentrations of *A*. *alternata* spores in form of standard dilutions, taken from samples free from both mycelia, spore fragments and other spores. To produce a standard curve, genomic DNA was extracted from a suspension of the *A*. *alternata* spores, grown and quantified as described in ‘Cultivation and quantification of *A*. *alternata* conidia as reference for qPCR’ section. The standard curve was generated by the Roche LightCycler 480 by plotting the ‘Crossing point’ (Cp) value for each dilution similar to Grinn-Gofroń et al. (2016) and Black et al. (2013). Mean spore concentrations mL^−1^ were generated using the second derivative maximum method in the LightCycler 480 software by measuring the Cp at which the fluorescent signal generated by amplification reaches the maximum second derivative. In second derivative maximum analysis, the number of spores mL^−1^ is calculated by polynomial regression of Cp against cycle number.

Mean air spore concentrations were determined against the standard curve generated from the cultured *A. alternata* spores. In both instances, concentrations were measured as the number of spores mL^−1^ due to suspension of spores in solution prior to DNA extraction. After quantification, the number of spores m^−3^ air was calculated from the number of spores mL^−1^. The cyclone samples 16.5 L air min^−1^ and operates for 24 h, therefore sampling 23.76 m^3^ air day^−1^, or 166.32 m^3^ air week^−1^. For simplicity, we will onwards term the DNA spore extraction as *A*. *alternata* spore equivalents; calculated as spores m^−3^ = spores mL^−1^/166.32 m^3^ as it should be noted that the DNA extraction and amplification of the air samples will detect both airborne spores and spore/hyphal fragments, whilst the reference samples that were quantified using a haemocytometer did not contain any spore/hyphal fragments. Since 2 μL of the eluted 100 μL (1/50th) DNA of the air samples was used in the qPCR reactions, the weekly air sample (166.32 m^3^) was divided by 50 = 3.326. Therefore, the weekly mean spore concentration was obtained by dividing the mean spore concentration from the LightCycler by 3.326.

### Meteorological data

Two Campbell Scientific meteorological stations were established at Worcester and Lakeside to provide half-hourly meteorological data for the period 2017–2018. Worcester meteorological station was co-located with the Burkard 7-day and cyclone samplers whilst Lakeside Container meteorological station was located 310 m away from the samplers. There was a gap in meteorological data from January 2016 to July 2017 before the acquisition of the Campbell Scientific meteorological instruments. The gap in data was filled with hourly meteorological data obtained from the nearest (20 km away) UK Met station (Pershore Weather Station; MET Office, UK). This was after verifying that relevant weather data from Pershore had a high correlation with Lakeside and Worcester weather data, similar to Skjøth et al. (2016).

The half-hourly and hourly weather data were independently averaged to provide weekly meteorological data for each meteorological station to match the weekly observations of *Alternaria* spp. and *A. alternata* spores. Selected meteorological parameters including mean air temperature (°C), pressure (hPa), relative humidity (%), solar radiation (W/m^2^), rain (mm), wind speed (m/s), wind direction (°), leaf wetness (minutes) and dew point (°C) were extracted for further analyses.

### Crop harvest data

To investigate the effect of crop harvesting on *Alternaria* spp. and *A. alternata* spore concentrations, reports of weekly crop harvest data were downloaded from the Agriculture and Horticulture Development Board (AHDB; https://ahdb.org.uk/cereals-oilseeds/gb-harvest-progress) and examined, similar to Apangu et al. (2020). AHDB is a statutory levy board and summarises the weekly harvest progress reports for specific regions of Great Britain that are supplied by independent agronomists weekly. The report covers crops such as winter wheat, winter oilseed rape, winter barley, spring wheat and spring barley. The crop harvest reports from the West Midlands region during the period from July to September of 2016 to 2018 were examined to explain the high spore concentrations during the harvest periods-starting with weeks when > 5% of the crops were harvested. To obtain the crop harvest data within 30 km (Avolio et al. 2008) from Worcester and Lakeside Container samplers, the percentage weekly crop harvest data (from AHDB) were multiplied with the land cover data for each crop above, similar to Apangu et al. (2020). The land cover for crops (in ha), produced by the UK Centre for Ecology and Hydrology (https://www.ceh.ac.uk/), was downloaded from the EDINA digimap website (https://digimap.edina.ac.uk/environment) and analysed using Spatial Analyst tool of ArcGIS 10.6.

### Statistical analyses of data

The descriptive summaries included start and end of sampling, weekly mean spore concentrations, peak week, peak spore concentration, total spores per site and days with daily spore concentrations above 100 spores m^−3^ (hereafter high days). For statistical analyses, the Shapiro –Wilk significance test was applied to test for normality of the spore data, similar to Kulik et al. (2015). Spearman’s rank correlation test was chosen after confirming from the Shapiro–Wilk significance test that the spores were not normally and linearly distributed. Spearman’s rank correlation test was performed between meteorological parameters observed at Worcester and Lakeside Container and weekly mean spore concentrations of *Alternaria* spp. and *A. alternata*, similar to Olsen et al. (2019) and Grinn-Gofroń et al. (2018). The same correlation analysis was performed between weekly mean *A*. *alternata*/*Alternaria* spp. spore concentrations and crop harvest data. The Spearman’s correlation test was applied to the logarithmically transformed weekly mean *A*. *alternata* and *Alternaria* spp. spore values to determine any relationship between them, similar to previous studies (Ding et al. 2021; Grinn-Gofroń et al. 2016; Pavón et al. 2012). The Wilcoxon signed-rank test was performed to compare the weekly mean *A*. *alternata* and *Alternaria* spp. spore concentrations and the days with daily mean spore concentration above 100 spores m^−3^ of air (clinical/high days) observed at Worcester and Lakeside Container, similar to previous studies (Kasprzyk and Worek 2006; Trigo et al. 2000). Multiple linear regression was performed to determine the most significant meteorological/harvesting variables contributing to airborne *A*. *alternata* spore equivalents and *Alternaria* spp. spore concentrations, as multiple linear regression is a vital technique to explain complex relationships in fungal spore concentrations (including *Alternaria*) in aerobiology (Martinez-Bracero et al. 2022). *Alternaria* spore data was transformed using a logarithmic function [(No. spores m^−3^) + 1] to obtain a normal distribution of the spore data prior to the multiple regression analyses, similar to previous studies (Angulo-Romero et al. 1999; Recio et al. 2012). All statistical analyses were performed in R v.4.1.3 (R Core Team 2022).

## Results

### Comparison of *Alternaria* spp. with *A. alternata* spore concentrations

*A*. *alternata* had a similar distribution pattern to *Alternaria* spp. spores and simultaneously peaked at both the rural site (Lakeside Container) and urban site (Worcester) in 2016 (Fig. 1a). Moreover, there was a strong and positive linear relationship between *Alternaria* spp. spore concentrations and *A*. *alternata* spore equivalents at Lakeside Container (*r* = 0.77, *p* = 0.003) and Worcester (*r* = 0.95, *p* < 0.001) in 2016 (Fig. 2a and b). However, despite the positive linear relationship (Fig. 2c–f) in both sites except in Worcester in 2017, *A*. *alternata* varied with *Alternaria* spp. spores in distribution and spatio-temporal pattern in 2017 (Fig. 1b) and 2018 (Fig. 1c) and they moderately-to-weakly correlated at Lakeside Container [2017 (*r* = 0.42, *p* = 0.16), 2018 (*r* = 0.5, *p* = 0.14)] and Worcester [2017 (*r* =  − 0.38, *p* = 0.25), 2018 (*r* = 0.14, *p* = 0.69)]. *A*. *alternata* spore concentrations peaked in July and August whilst *Alternaria* spp. spores peaked only in August at both the rural (Lakeside Container) and urban (Worcester) sites throughout the sampling periods.

**Fig. 1a:**
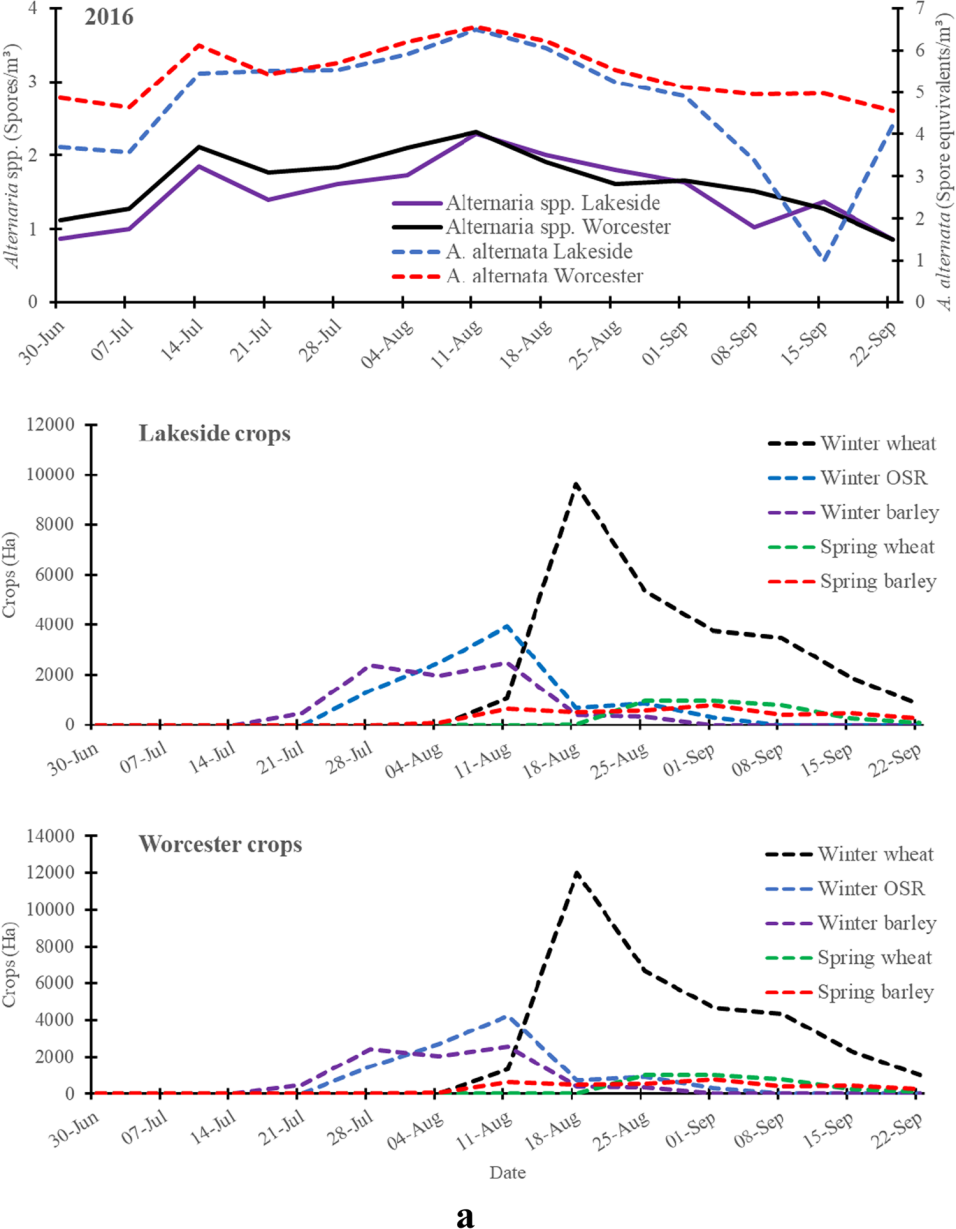
Spatio-temporal variation of the Log transformed weekly mean *A*. *alternata* (qPCR) and *Alternaria* spp. (optical microscopy) spore concentrations detected at Lakeside Container (rural site) and Worcester (urban site) during the same period of harvesting of the cereals and oilseed rape in 2016 and crop areas of main crops grown in a 30 km radius area centred on each of the Lakeside Container or Worcester sampling sites

**Fig. 1b:**
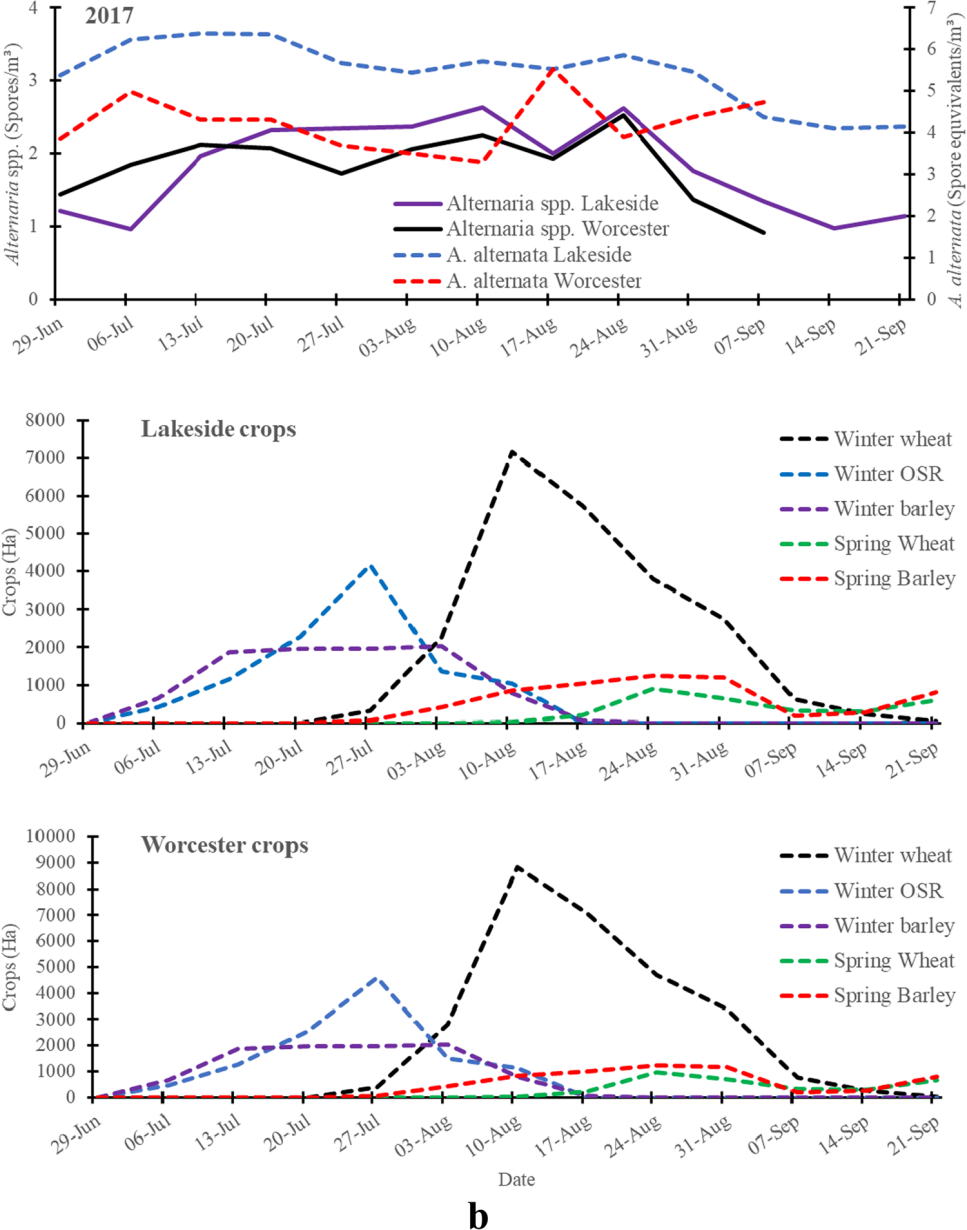
Spatio-temporal variation of the Log transformed weekly mean *A*. *alternata* (qPCR) and *Alternaria* spp. (optical microscopy) spore concentrations detected at Lakeside Container (rural site) and Worcester (urban site) during the same period of harvesting of the cereals and oilseed rape in 2017 and crop areas of main crops grown in a 30 km radius area centred on each of the Lakeside Container or Worcester sampling sites

**Fig. 1c:**
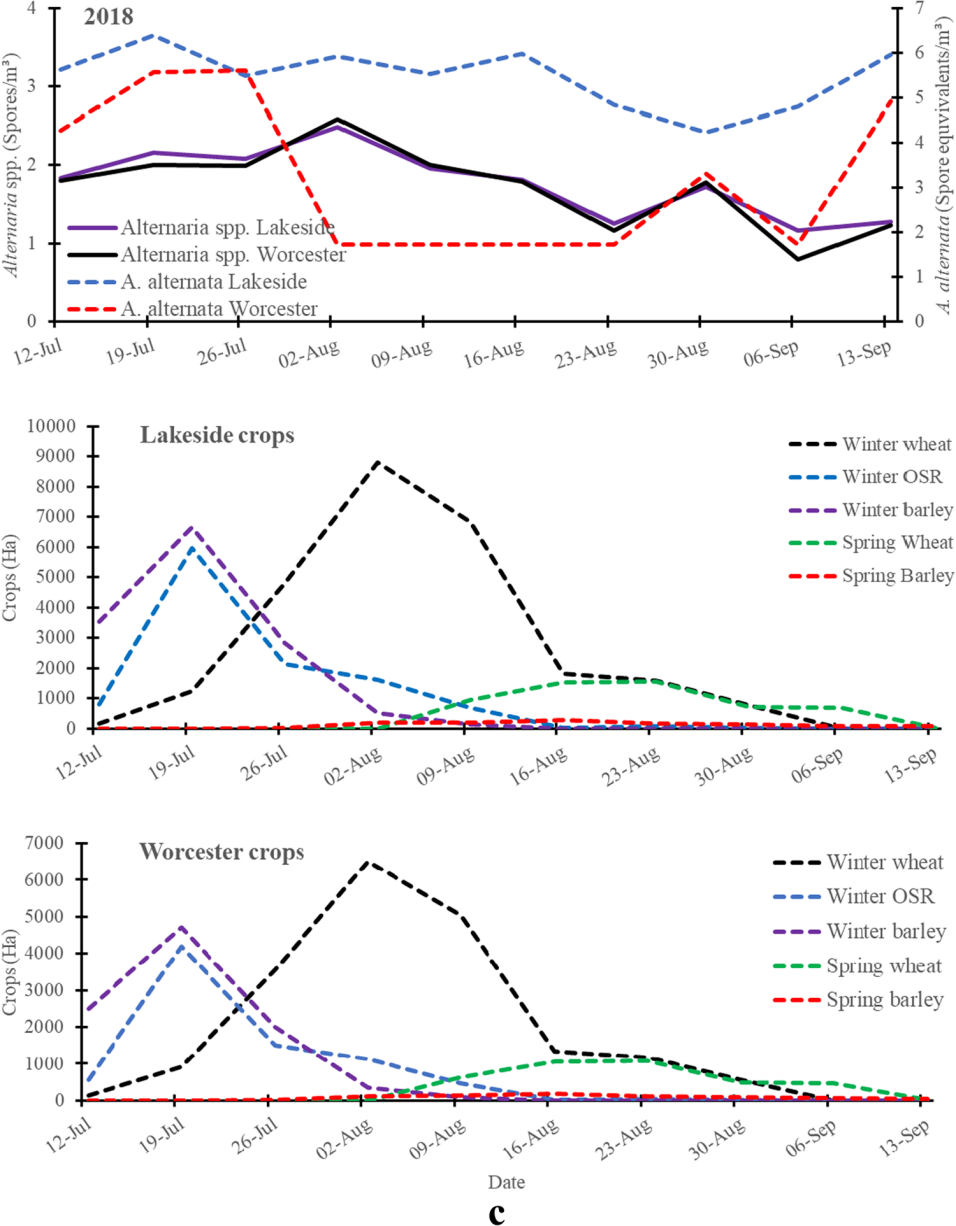
Spatio-temporal variation of the Log transformed weekly mean *A*. *alternata* (qPCR) and *Alternaria* spp. (optical microscopy) spore concentrations detected at Lakeside Container (rural site) and Worcester (urban site) during the same period of harvesting of the cereals and oilseed rape in 2018 and crop areas of main crops grown in a 30 km radius area centred on each of the Lakeside Container or Worcester sampling sites

**Fig. 2:**
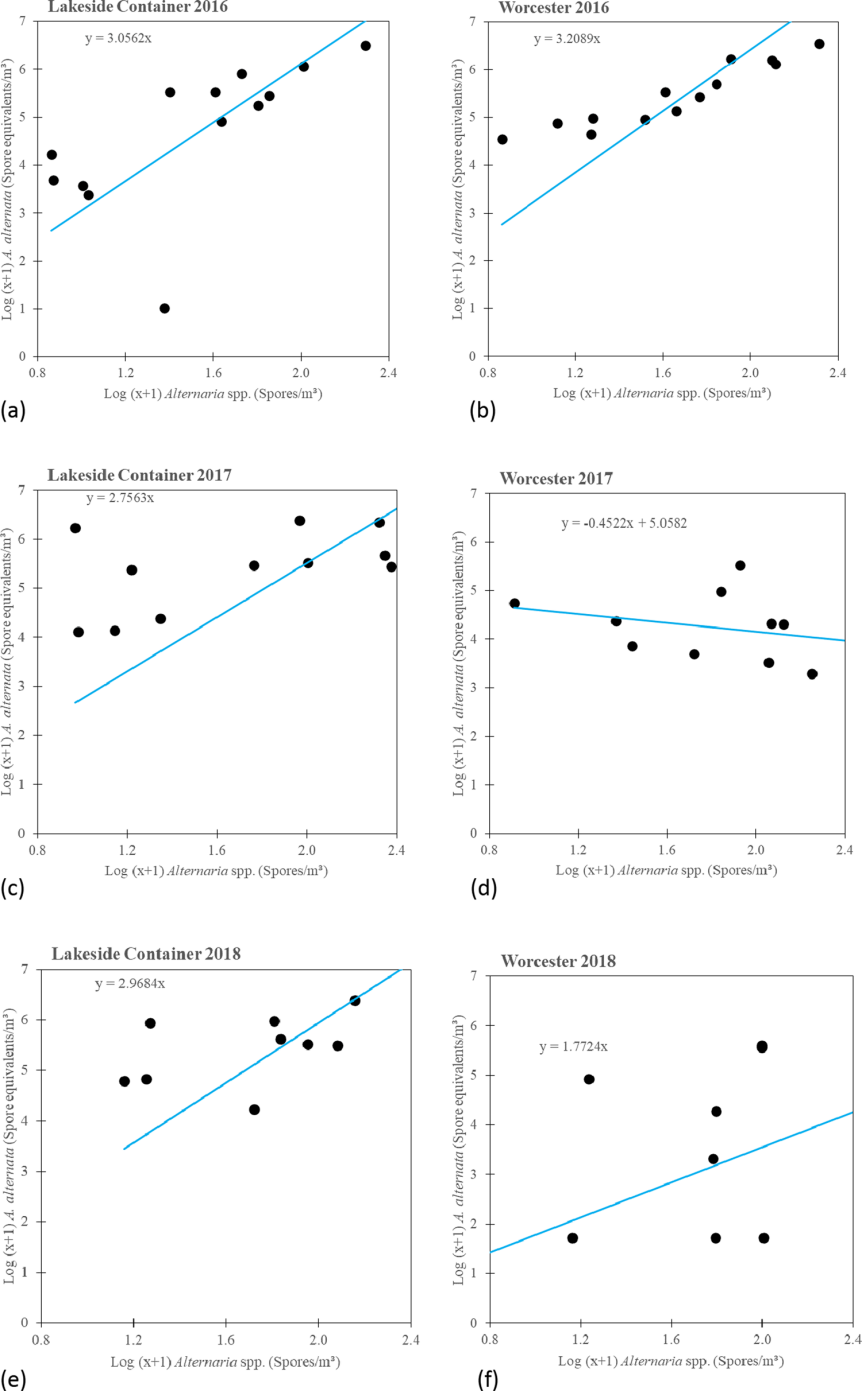
Spearman correlation coefficients between Log. transformed weekly mean *Alternaria* spp. and *A*. *alternata* spore equivalents collected at Lakeside Container (rural site) and Worcester (urban site) in 2016 (**a**, **b**), 2017 (**c**, **d**), and 2018 (**e**, **f**)

Total and peak *A*. *alternata* spore concentration equivalents, based on collections with the cyclones and qPCR, were considerably higher than those of *Alternaria* spp. based on the collection with the 7-day Burkard trap and the optical microscopy at both the rural (Lakeside Container) and urban (Worcester) sites, except in the period late July to 16 August 2018 (Fig. 1a–c; Table S1). The peak and total spore concentrations of *A*. *alternata* spores were dominated by the rural site in 2017 and 2018 (Table S1). Meanwhile, the peak and total spore concentrations of *Alternaria* spp. spores were dominated by the urban site in 2016 and 2018.

Wilcoxon signed-rank statistical analyses of the weekly mean *A*. *alternata* spore equivalents revealed that the rural site was significantly different from the urban site during the 2016 (*p* = 0.002), 2017 (*p* = 0.005) and 2018 (*p* = 0.014) spore seasons. Moreover, analyses of the high days (clinically important days) also showed that the rural site significantly varied from the urban site in 2017 (*p* = 0.010). However, there was no significant difference in the weekly mean *Alternaria* spp. spore concentration between the two sites in 2016 (*p* = 0.077), 2017 (*p* = 0.067) and 2018 (*p* = 0.646). Similarly, the sites showed no statistical difference in the high days in 2016 (*p* = 0.910) and 2018 (*p* = 0.932).

Using optical microscopy, *Alternaria* spp. spores were only identified to genus level whilst qPCR detected the specific *A*. *alternata* spores from the air samples within the recommended quantification cycle value of < 40 (Fig. S2a) and with high efficiency (2.084; Fig. S2b). The primer pair, as revealed by the melt curve temperature (Fig. S2c), was sufficiently specific and its sequences matched 100% with that of *A*. *alternata* (accession numbers OM422689.1 and OM422688.1).

### *A*. *alternata* spore equivalents and *Alternaria* spp. spore concentrations during crop harvesting

Overall, analysis of the weekly crop harvest data showed that *A*. *alternata* spore equivalents and *Alternaria* spp. spore concentrations considerably and significantly increased during the harvesting of the cereals and oilseed rape (Fig. 1a–c; Table S2**)**. The minor peak of *A*. *alternata* and *Alternaria* spp. spores at both the rural site and urban site on 14 July 2016 was observed when < 5% of the crops were harvested. However, the major peak of *A*. *alternata* and *Alternaria* spp. spores (11 August 2016) was observed when winter wheat, winter barley and winter oilseed rape were harvested in considerable amounts at both sites in 2016 (Fig. 1a). Harvesting of winter wheat increased *Alternaria* spp. spore concentrations at both Lakeside Container and Worcester and* A*. *alternata* at Lakeside Container in 2017 (Fig. 1b) and 2018 (Fig. 1c). Meanwhile, harvesting of mainly winter barley and winter oilseed rape increased both *Alternaria* spp. and *A*. *alternata* spore concentrations at both Worcester and Lakeside Container in 2017 and 2018, especially around the peak periods.

Crops harvested significantly correlated with *A*. *alternata* spore equivalents and *Alternaria* spp. spore concentrations (Table S2). However, winter oilseed rape and winter barley were the significant and most frequent contributors to airborne *A*. *alternata* spore equivalents at any of the two sites; meanwhile, simultaneous harvesting of all the crops during a week (total weekly harvest) and of winter wheat were the significant and greatest contributors to *Alternaria* spp. spore concentrations.

The multiple linear regression found that none of the crops had a significant effect on *A*. *alternata* spore equivalents or *Alternaria* spp. spore concentration (Table 1). However, the model showed winter barley and total weekly harvest as potential candidates that could have contributed to *A*. *alternata* spore equivalents and *Alternaria* spp. spore concentrations. Overall, the model showed that crop harvesting contributed 13.2% (R^2^ = 0.132) and 46.7% (R^2^ = 0.467) of the variation in *A*. *alternata* spore equivalents and *Alternaria* spp. spore concentrations respectively.

**Table 1:** Model statistics and significance levels for the multiple linear regression of log-transformed (a) *A*. *alternata* spore equivalents and (b) *Alternaria* spp. spore concentrations in relation to the weekly crop harvesting for the 3 years (2016–2018) and two locations (Lakeside Container and Worcester)

Model performance: Adjusted R^2^: (a) 0.132, (b) 0.467. Model statistics: ANOVA (Type II)					
Model variable	Model statistics				
	Sum Sq	Df	*F* value	*P* value	Significance
(a)					
Winter wheat	0.744	1	0.493	0.485	ns
Winter OSR	0.651	1	0.431	0.514	ns
Winter barley	5.573	1	3.692	0.059	·
Summer wheat	1.860	1	1.232	0.271	ns
Summer barley	2.028	1	1.343	0.251	ns
Total harvest	1.640	1	1.087	0.301	ns
Residuals	95.108	63	na	na	na
(b)					
Winter wheat	0.016	1	0.123	0.727	ns
Winter OSR	0.012	1	0.097	0.756	ns
Winter barley	0.473	1	3.706	0.059	·
Summer wheat	0.157	1	1.231	0.271	ns
Summer barley	0.169	1	1.327	0.254	ns
Total harvest	0.406	1	3.183	0.079	·
Residuals	8.039	63	na	na	na

Significance: ***P < 0.001; **P < 0.01; *P < 0.05; ·*P* < 0.1; *P* > 0.1 ns

*Df* degree of freedom, *na* not applicable, *OSR* oilseed rape, *Total Harvest* total weekly harvest

### Relationship between meteorological parameters and *Alternaria* spp./*A. alternata* spore concentrations

Overall, the peak *A*. *alternata* and *Alternaria* spp. spore concentrations were observed when high relative humidity (above 70%) and air temperature (above 15 °C) and low rain (below 20 mm) were recorded at both Lakeside Container and Worcester in the 3 years of observation (Fig. S3a–c). However, there was an anomaly in 2017 where high *A*. *alternata* spore concentration was observed at Worcester when a high amount of rain (above 40 mm) was recorded, whilst *Alternaria* spp. spore concentration remained low at both sites.

Several meteorological variables significantly correlated with *Alternaria* spp. spore concentrations and *A*. *alternata* spore equivalents at either the rural (Lakeside Container) or the urban (Worcester) site. Spearman’s correlation test (Table S3) found that wind direction correlated with *A*. *alternata* spore equivalents at only the rural site in 2017. Air temperature significantly correlated with either *Alternaria* spp. spore concentration or *A*. *alternata* spore equivalents at either the rural or urban site during the three years of observation. Dew point temperature significantly corrected with both *Alternaria* spp. spore concentration and *A*. *alternata* spore equivalents at the rural site in 2017 and 2018 whilst it correlated with *Alternaria* spp. spore concentration at the urban site in 2018. Relative humidity was significantly and inversely correlated with *A*. *alternata* spore equivalents at the rural site in 2016 and 2017 and the urban site in 2018. Meanwhile, relative humidity was significantly and inversely correlated with *Alternaria* spp. spore concentration at the rural site in 2017 and 2018. Solar radiation was significantly correlated with *Alternaria* spp. spore concentration at the rural site in 2017 and 2018 and at the urban site in 2018, whereas solar radiation significantly correlated with *A*. *alternata* spore equivalents at only the rural site in 2017. Leaf wetness significantly correlated with only *A*. *alternata* spore equivalents at the rural site in 2017 and 2018. Overall, the air temperature was the weather variable that was most frequently associated with either *A*. *alternata* spore equivalents or *Alternaria* spp. spore concentration at either the rural or urban site.

Multiple linear regression found that wind direction and air temperature significantly contributed to the airborne *A*. *alternata* spore equivalents (Table 2(a)); meanwhile, wind direction, wind speed, dew point temperature and relative humidity significantly increased *Alternaria* spp. spore concentrations (Table 2(b)) at all the sites. Overall, the model showed that weather contributed 23.9% (R^2^ = 0.239) and 46.3% (R^2^ = 0.463) of the variation in *A*. *alternata* spore equivalents and *Alternaria* spp. spore concentrations respectively.

**Table 2:** Model statistics and significance levels for the multiple linear regression of log-transformed (a) *A*. *alternata* spore equivalents and (b) *Alternaria* spp. spore concentrations in relation to meteorological variables for the 3 years (2016–2018) and two locations (Lakeside Container and Worcester). Solar radiation and leaf wetness were not included in the model because they were not observed for both sites for all the years of observation

Model performance: adjusted R^2^: (a) 0.239, (b) 0.463. Model Statistics: ANOVA (Type II)					
Model variable	Model statistics				
	Sum Sq	Df	*F* value	*P* value	Significance
(a)					
Wind direction	8.124	1	6.138	0.016	*
Wind speed	4.063	1	3.070	0.085	·
Atmospheric pressure	3.172	1	2.396	0.127	ns
Temperature	6.337	1	4.787	0.032	*
Dew point	4.989	1	3.769	0.057	·
Precipitation	0.001	1	0.001	0.978	ns
Relative humidity	2.333	1	1.763	0.189	ns
Residuals	82.068	62	na	na	na
(b)					
Wind direction	3.442	1	26.745	0.000	***
Wind speed	1.406	1	10.922	0.002	**
Atmospheric pressure	0.093	1	0.723	0.398	ns
Temperature	0.265	1	2.057	0.157	ns
Dew point	0.614	1	4.773	0.033	*
Precipitation	0.003	1	0.024	0.878	ns
Relative humidity	0.699	1	5.435	0.023	*
Residuals	7.979	62	na	na	na

Significance: ***P < 0.001; **P < 0.01; *P < 0.05; ·*P* < 0.1; *P* > 0.1–ns. *Df* degree of freedom, *na* not applicable

## Discussion

### Relationship between airborne *A. alternata* and *Alternaria* spp. spores

We partly accepted the hypothesis that the high *Alternaria* spore concentrations in 2016 were mainly contributed by *A*. *alternata*. The high *A*. *alternata* spore equivalents (measured with qPCR) compared to *Alternaria* spp. spore concentrations (measured with microscopy) is partly attributed to the fact that qPCR estimates spore numbers based on the quantification of the total genomic DNA in air samples, which includes the spores, hyphae and their fragments (Zeng et al. 2006). Fungal fragments can significantly exceed intact spores by concentration (Green et al. 2005). Aerosolization experiments have shown a 300-fold increase in the fungal fragments (Górny et al. 2002). Grewling et al. (2020) reported a low amount of the allergen Alt a1 in spore or hyphal fragments compared to intact *Alternaria* spores. However, it has been suggested that hyphal fragments from *A*. *alternata* contain few or no Alt a 1 allergens (Saha et al. 2022), whilst other allergens may be present. Pashley et al. (2012) found a discrepancy between optical microscopy and the DNA-based approach in the detection of *Botrytis* spores in air samples and attributed this to DNA from teleomorphs and hyphal fragments, since DNA-based analysis does not distinguish between DNA from spores or hyphae. Consequently, these fungal fragments may rapidly increase concentrations of allergenic and pathogenic fungal particles in the air, especially in agricultural areas that undergo crop combine-harvesting (Ammour et al. 2020; Lee and Liao 2014; Olsen et al. 2019; Tomlin et al. 2020). Importantly, as in our study, Pashley et al. (2012) used a cyclone sampler for the DNA extraction and a Hirst type volumetric trap for the microscopic counting of spores, both from Burkard. According to the manufacturer, the cyclones will be efficient in collecting small particles, whilst the same is not stated for the Hirst type volumetric trap. Therefore, it is likely that the high levels of *A*. *alternata* detected by the qPCR assays in all the air samples collected with the cyclone sampler from both the rural and urban sites could have included fragments of *A*. *alternata* spores or high amounts of hyphae. Fragments of *Alternaria* spores or hyphae can contain both DNA and several allergens and the hyphae may grow when they meet a susceptible plant host (Rosemond and Kramer 1971). They can penetrate further into the lungs compared with large spores that are intact and are in some environments suspected to be present in much higher concentrations than intact spores (Green et al. 2006). Future studies should consider the quantification of fungal fragments and hyphae using both qPCR and immunological approaches using samplers that efficiently capture this small particle fraction to identify their contribution to the dispersal of aeroallergens and pathogens.

The dominance of *A*. *alternata* spores over other *Alternaria* species spores is partly attributed to weather and crop harvesting, since wind direction, air temperature and potentially harvesting of winter barley were found to significantly contribute to *A*. *alternata* spore equivalents. Previous studies also found similar results regarding the positive effect of air temperature, wind speed, relative humidity and precipitation on airborne *Alternaria* spore concentrations (Angulo-Romero et al. 1999; Rodríguez-Rajo et al. 2003; Recio et al. 2012; Sidel et al. 2015; Ščevková et al. 2019; Fagodiya et al. 2022). However, none had included crop harvest data in the model analyses. This is the first study that reports on the contribution of crop harvesting to *A*. *alternata* spore concentrations.

During crop harvesting, there are harvest intervals when crops for consumption cannot be sprayed with fungicides and growers rely on good weather to proceed with harvesting, as was demonstrated with the significant correlations between *A*. *alternata*/*Alternaria* spp. spore concentrations and weather/crop harvest variables in our study. The 2016 results show that the temporal variations of *A*. *alternata* spore concentrations can be used as a proxy to predict other pathogenic and allergenic *Alternaria* species in an environment. To our knowledge, this is the first study that used the DNA of *A*. *alternata* spores to estimate the spore concentration of other *Alternaria* species in the air. *A*. *alternata* is considered a world-wide pathogen in agriculture (e.g. Kgatle et al. 2019). It is also found to be the most prevalent indoor *Alternaria* species in the USA (Woudenberg et al. 2015) and unlike many other *Alternaria* species* A*. *alternata* is not host specific. Therefore, it may be hypothesized that *A*. *alternata* may be a reasonable proxy to predict the development of a large range of pathogenic and allergenic *Alternaria* species within large parts of the world. *A*. *alternata* should therefore be a target for further investigations and in particular forecasting models.

An anomaly of high *A*. *alternata* and low *Alternaria* spp. spore concentrations during or after rain was observed in 2017. The low *Alternaria* spp. spore concentration is attributed to the scavenging effect of rain on larger bioaerosols such as intact *Alternaria* spp. spores including *A*. *alternata* (O’Connor et al. 2014), whilst spore fragments or small hyphae may escape this scavenging due to their smaller particle size (Berthet et al. 2010; Miki 2023). Raindrops, through spore dislodgement and with the support of wind, could have dispersed the already existing large amounts of *A*. *alternata* spores and spore/hyphal fragments on soil and vegetation or those in raindrops as was observed with fungal and bacterial spores (Joung et al. 2017; Zhai et al. 2018). The high *A*. *alternata* spore concentration after rain is due to sporulation and faster growth of the spores immediately after deposition on the right host and in a conducive environment, e.g. humidity (Humpherson-Jones and Phelps 1989; Hjelmroos 1993). Blagojević et al. (2020) showed that *A*. *alternata* spores grow faster than other common *Alternaria* species, e.g. *A*. *brassicae*, *A*. *brassicicola* and *A*. *japonica* over a wide range of temperature. Despite the complicating factors, then the most probable explanation of the anomaly seen in 2017 is how the scavenging effect by rain can efficiently remove larger intact spores, whilst a fraction of the smaller spore fragments and hyphae can remain airborne.

The model results suggest that, apart from its wide host range, the weather and crop harvesting, there are other significant and unknown underlying variables that were contributing to the dominance and abundance of *A*. *alternata* spores or spore/hyphal fragments over other *Alternaria* spp. spores in 2016. Previous studies also showed that the type of fungal species, weather conditions and mechanical disturbance of the spore source, could determine the simultaneous release and dominance of one type of species over others (Grewling et al. 2020; Madsen et al. 2016). Apart from the dominance and abundance of *A*. *alternata* spore equivalents, this study also demonstrated that the spores of *A*. *alternata* and other *Alternaria* species can be simultaneously released from their sources and have a similar distribution pattern when the weather, i.e. air temperature and relative humidity, is suitable for them all and crop harvesting takes place. This suggests the natural co-existence of *A*. *alternata* with other *Alternaria* species when they have similar growth and environmental conditions that facilitate their simultaneous dispersal. This complements and extends findings in Hanson et al.’s (2022) study, also undertaken in this rural – urban study region, that found several *Alternaria* species to be present at the same time. Audenaert et al. (2009) and Xu et al. (2005) also found positive co-existence patterns among *Fusarium* species, i.e. *F*. *poae*, *F. avenaceum* and *F*. *culmorum.* The simultaneous release of *A*. *alternata* and other *Alternaria* species also demonstrates the extent of fungal diversity in the air and its implication on human health and agriculture/forestry. Sensitised people can be simultaneously exposed to different fungal species in the air (Madsen et al. 2016). Hong et al. (2005) showed that 37 different *Alternaria* species including *A*. *alternata*, *A*. *brassicicola*, *A*. *tenuisima* and* A*. *infectoria* contain the major *Alternaria* allergen Alt a 1. Significantly high fungal diversity associated with an increase in symptoms of respiratory infections was observed in schoolchildren in fungal-infested and moisture-damaged schools (Meklin et al. 2005). Furthermore, different *Alternaria* species including *A*. *alternata*, *A*. *solani* and *A*. *infectoria* were isolated from closely located potato fields, signifying the potential of simultaneous infection by different *Alternaria* species for total yield loss in crops (Leiminger et al. 2014; Belosokhov et al. 2017). Knowledge of the co-existence of pathogenic and allergenic fungal communities is important for effective disease management, forecasting and allergy prevention.

### Spatio-temporal variation between *A. alternata* and *Alternaria* spp. spore concentrations

We partly rejected the hypothesis that the high *Alternaria* spore concentrations in 2017 and 2018 were mainly contributed to by *A*. *alternata.* This suggests that other *Alternaria* species were the most dominant and in higher abundance than *A*. *alternata* during the spore seasons of 2017 and 2018. Weather played a significant role in the dominance and high abundance of other *Alternaria* species. Variation in the spatio-temporal pattern and peak weeks of *Alternaria* spp. compared to *A*. *alternata* spores is in line with Pashley et al. (2012), who attributed the differences to weather variables such as precipitation, relative humidity and wind speed. The spatio-temporal variations and non-significant relationships suggest that, besides *A*. *alternata*, there were other *Alternaria* species in the urban (Worcester) and rural (Lakeside Container) sites with differing spatio-temporal patterns that were being detected by microscopy. Studies of spatio-temporal patterns in *Alternaria* are rare. Ding et al. (2021) studied the spatio-temporal pattern of *A*. *alternata* and *A*. *solani* in potato fields in Wisconsin, USA, and found that *A*. *alternata* spores were always dispersed in late June whilst *A*. *solani* started later and both varied spatially, owing to differences in host ranges of the two species and variation in abundance of inoculum at the different potato fields. Although the urban (Worcester) and the rural (Lakeside Container) sites generally had ecosystems with similar hosts (grasslands and farmland; Apangu et al. 2020; Rowney et al. 2021), differences in the ecosystem conditions between the two sites could have caused the spatio-temporal variations of *Alternaria* spp. This was demonstrated by Reis and Boiteux (2010) who found that, although *A*. *brassicae* and *A*. *brassicicola* are both major pathogens of the Brassicaceae, *A*. *brassicicola* was more prevalent in *Brassica oleracea* complex whilst *A*. *brassicae* infects mostly the *B*. *rapa* complex, thus attacking the crops at different times of the growing season. Similarly, Schiro et al. (2018) found that *Alternaria* spp. spore distribution and abundance were strongly influenced by local ecological conditions within a topographically heterogeneous field of wheat.

The spatio-temporal variations in this study in 2017 and 2018 also demonstrate a lack of co-existence between *A*. *alternata* and other *Alternaria* species on the same hosts. A lack of co-existence and co-dispersal between fungal species may occur due to competition for space, antibiosis, differences in growth conditions and dissimilarities in response to environmental requirements (Nicolaisen et al. 2014). Antagonistic relationships due to antibiosis have been found within *Alternaria* spp. and between *Alternaria* spp. and other fungal species, e.g. *Fusarium culmorum*, *Botrytis cinerea* and *Cladosporium herbarum* (Kilic et al. 2010; Lević et al. 2012; Liggitt et al. 1997; Nicolaisen et al. 2014). A consequence of the non-significant relationship and antagonistic spatio-temporal pattern between *A*. *alternata* and *Alternaria* spp. spore concentrations is that allergy/asthma patients and farmers/foresters will observe many extended days of clinical and pathological significance. In general, there will be spatio-temporal differences in sporulation and release of spores from different *Alternaria* species into the atmosphere because different species of *Alternaria* are located on different substrates and will be at different stages of growth and in competition with other microbes especially during crop senescence and will respond differently to weather cues.

### Difference between *A. alternata* and *Alternaria* spp. spore concentration in nearby rural and urban sites

The statistically similar *Alternaria* spp. spore concentrations at both sites was expected, since the two sites are closely located and generally have similar vegetation cover and crop production near the spore traps. Antón et al. (2021) found similar results for two samplers that were located 1.4 km apart. However, statistical analyses revealed that the rural site had significantly (*p* < 0.05) different *A*. *alternata* spore concentrations from the urban site in all the 3 years of spore observation. Moreover, the two sites also significantly varied from each other during the high days in 2017. Similarly, previous studies that examined *Alternaria* spp. spore concentrations between nearby (< 10 km) rural and urban sites also found statistically significant difference in spore concentrations and diversity between such sites (Hanson et al. 2022; Kasprzyk and Worek 2006; Lin et al. 2018; Oliveira et al. 2009). The variation between such nearby sites could be attributed to the difference in the density of crops near the traps, their spore emission potential (Apangu et al. 2020) and the effect of mechanical farming operations and local weather on *Alternaria* sporulation and spore release (Schiro et al. 2018; Skjøth et al. 2012, 2016). In examining the *A*. *alternata* pattern for two nearby (~ 7 km apart) sites, this study extends the previous studies of closely located sites that focused on only *Alternaria* spp. spores in general and suggests that care should be taken when comparing *Alternaria* spp. spores from several sites for application in clinical or pathological studies as the species composition and abundance can vary between nearby sites.

### Comparison of optical microscopy with qPCR method in detecting *Alternaria* spp. and *A. alternata* spores

High concentrations of *A*. *alternata* spore equivalents from the qPCR, compared to the *Alternaria* spp. concentrations using microscopy, may partly be attributed to the fact that optical microscopy cannot always discriminate the different *Alternaria* species (Luo et al. 2007), whereas the DNA analysis can. Moreover, Parker et al. (2014) and Pizolotto et al. (2022) found very high concentrations of *Sclerotinia sclerotiorum* and *Erysiphe betae*, respectively, using qPCR in contrast to microscopy. Differences may also be attributed to variation in the sampling efficiencies of Burkard cyclones (16.5 L/min) and spore traps (10 L/min), which vary with particle size. The Burkard multi-vial cyclone sampler has a high sampling efficiency (93.82–100%), especially for small-sized (≤ 1 µm) bioaerosols (De Linares et al. 2022; Humbal et al. 2018; West et al. 2008) compared with the Burkard 7-day sampler that is most efficient for collecting particles with an aerodynamic diameter of > 5.2 µm (Ščevková and Kovac 2019). Almaguer et al. (2020) analysed fungal fragments alongside mature spores and found that hyphal fragments of *Cladosporium* and *Leptosphaeria* were abundant in the air and had no diurnal pattern. Their abundance and lack of diurnal pattern suggest that they are constantly abundant (day and night) in the air and hence can be captured by the cyclones. This puts further emphasis on the need to quantify fungal fragments in the air.

## Conclusion

In summary, the study found high spore concentration of *A*. *alternata* spore equivalents (using qPCR) compared to *Alternaria* spp. (from optical microscopy), and this was likely due to spores and spore/hyphal fragments. The spore numbers should be used with caution because in most cases a fraction of the spore count obtained with the optical microscope originates from other *Alternaria* species. Besides *A*. *alternata*, many other *Alternaria* species remain unidentified in spore counts by microscopy. Furthermore, spore material may be present as fragments with the potential to penetrate further into the lungs. These fragments are not counted and are also less likely to be captured by the impaction technique used to deliver material on microscope slides. The spatio-temporal invariability in 2016 and variations in 2017 and 2018 in the distribution of *A*. *alternata* from other *Alternaria* species were due mainly to weather variations, especially mean air temperature and relative humidity and harvesting of cereals and oilseed rape. Future studies should examine such relationships between particular species of *Alternaria* and other pathogenic and allergenic fungal species, at a higher temporal resolution (less than a week) and with more emphasis on spore fragments. The rural site significantly varied from the urban site in *A*. *alternata* spore concentration even though they were closely located (approximately 7 km apart) and this was attributed to differences in local weather and harvesting activities.

## Supplementary Information

Below is the link to the electronic supplementary material.

Supplementary file1 (DOCX 1362 KB)

## Data Availability

All data generated or analysed during this study are included in this published article [and its supplementary information files].
