# Supplementary material for: Using qPCR and microscopy to assess the impact of harvesting and weather conditions on the relationship between Alternaria alternata and Alternaria spp. spores in rural and urban atmospheres
Source: Int J Biometeorol. 2023 May 16;67(6):1077–93. doi: 10.1007/s00484-023-02480-w (PMC10267013; doi:10.1007/s00484-023-02480-w)
Supplement: Supplementary file 1 — Supplementary file1 (DOCX 1362 KB) [file 484_2023_2480_MOESM1_ESM.docx]

**Electronic Supplementary Material: International Journal of Biometeorology**

**Using qPCR and microscope to assess the impact of harvesting and weather on *Alternaria alternata* and *Alternaria* spp. spores in rural and urban atmospheres**

**Godfrey Philliam Apangu ^1, *^. Carl Alexander Frisk ^2^. Beverley Adams-Groom. Geoffrey M. Petch. Mary Hanson ^3^. Carsten Ambelas Skjøth ^4^**

School of Science and the Environment, University of Worcester, Henwick Grove, WR2 6AJ, Worcester, UK.

^*^Corresponding author E-mail: godfrey.apangu@rothamsted.ac.uk


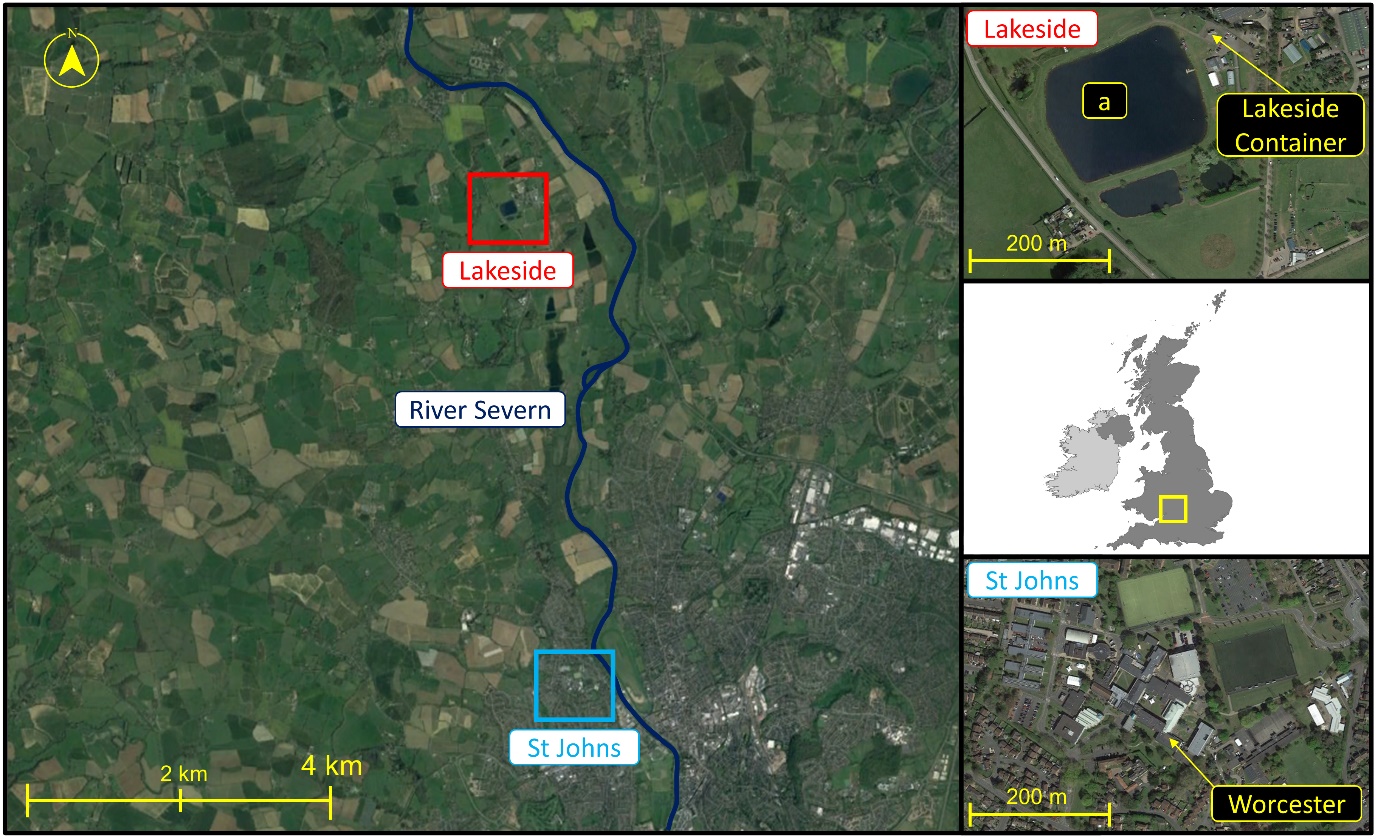


**Fig. S1** Showing the spore sampling and meteorological station sites of Lakeside Container and Worcester on the left. Also indicated on the map is the (a) Lake near Lakeside Container and River Severn (dark blue spiralling feature on left map). Lakeside and St Johns maps on the right are enlargements of their corresponding map sections on the left

(a)

(b)

**Fig. S2** (a) Amplification curve of the air samples during qPCR assay and (b) their corresponding standard curve showing the Log concentration of Spores mL ^-1^ against crossing point (Cp) determined by the second derivative maximum analysis on a LightCycler 480 (Roche Diagnostics)

**Fig. S2c** Melt curve analysis with the derivative of fluorescence plotted against temperature generated during the qPCR using the primer set for the specific detection of *A. alternata* spores from air samples (amplification at 87 ^0^C)

**Fig. S3a** Log. transformed weekly mean *A. alternata* and *Alternaria* spp. spore concentrations in the air of Lakeside Container (rural site) and Worcester (urban site) during weather [air temperature (Temp.; ^0^C), rain (mm) and relative humidity (RH; %)] changes in 2016

**Fig. S3b** Log. transformed weekly mean *A. alternata* and *Alternaria* spp. spore concentrations in the air of Lakeside Container (rural site) and Worcester (urban site) during weather [air temperature (Temp.; ^0^C), rain (mm) and relative humidity (RH; %)] changes in 2017. Note that Worcester spore data was only available up to 7 Sep 2017

**Fig. S3c** Log. transformed weekly mean *A. alternata* and *Alternaria* spp. spore concentrations in the air of Lakeside Container (rural site) and Worcester (urban site) during weather [air temperature (Temp.; ^0^C), rain (mm) and relative humidity (RH; %)] changes in 2018

**Table S1** Summary of the sampling periods, clinical (high) days, peak value and week, weekly spore mean and total spore collected at Lakeside Container and Worcester during 2016-2018 season

| **Year** | **Location** | **Spore type** | **Sampling start** | **Sampling end** | **High days** | **Peak value (Spores/m³)** | | **Peak week** | **Weekly mean (Spores/m³)** | | **Total spore (Spores/m³)** | |
| --- | --- | --- | --- | --- | --- | --- | --- | --- | --- | --- | --- | --- |
| 2016 | Lakeside Container | *Alternaria* spp. | 30-Jun | 28-Sep | 14 | 196 | 11-17-Aug | | 50 | 644 | |  |
|  |  | *A. alternata* | 30-Jun | 28-Sep | NA | 309,681 | 11-17 Aug | | 48,590 | 631,667 | |  |
|  | Worcester | *Alternaria* spp. | 30-Jun | 28-Sep | 17 | 205 | 11-17 Aug | | 64 | 835 | |  |
|  |  | *A. alternata* | 30-Jun | 28-Sep | NA | 351,774 | 11-17 Aug | | 74,548 | 969,122 | |  |
| 2017 | Lakeside Container | *Alternaria* spp. | 29-Jun | 13-Sep | 33 | 432 | 10-16 Aug | | 165 | 1,812 | |  |
|  |  | *A. alternata* | 29-Jun | 13-Sep | NA | 2,459,411 | 13-19 Jul | | 846,611 | 9,312,718 | |  |
|  | Worcester | *Alternaria* spp. | 29-Jun | 13-Sep | 24 | 333 | 24-30 Aug | | 103 | 1,131 | |  |
|  |  | *A. alternata* | 29-Jun | 13-Sep | NA | 333,734 | 17-23 Aug | | 52,222 | 574,438 | |  |
| 2018 | Lakeside Container | *Alternaria* spp. | 12-Jul | 19-Sep | 20 | 307 | 2-8 Aug | | 89 | 889 | |  |
|  |  | *A. alternata* | 12-Jul | 19-Sep | NA | 2,435,358 | 19-25 Jul | | 633,870 | 6,338,695 | |  |
|  | Worcester | *Alternaria* spp*.* | 12-Jul | 19-Sep | 21 | 380 | 2-8 Aug | | 89 | 894 | |  |
|  |  | *A. alternata* | 12-Jul | 19-Sep | NA | 396,873 | 19-25 Jul | | 87,085 | 870,851 | |  |

NA-Not Applicable

**Table S2** Spearman’s correlation coefficient between absolute weekly crop harvest data and the weekly mean *A. alternata/Alternaria* spp. spore concentrations for the periods 7 Jul-28 Sep 2016, 29 Jun-13 Sep 2017 and 12 Jul-19 Sep 2018

| **Location and year** | **Spore type** | **Winter wheat** | **Winter OSR** | **Winter barley** | **Spring wheat** | **Spring barley** | **Total weekly harvest** |
| --- | --- | --- | --- | --- | --- | --- | --- |
| Lakeside Container 2016 | *A. alternata* | 0.598* | 0.791* | 0.750* | 0.050 | 0.356 | 0.809* |
|  | *Alternaria* spp. | 0.662* | 0.429 | 0.433 | 0.370 | 0.562 | 0.697* |
| Worcester 2016 | *A. alternata* | 0.532 | 0.623* | 0.705* | 0.114 | 0.381 | 0.729* |
|  | *Alternaria* spp*.* | 0.498 | 0.580* | 0.759* | 0.013 | 0.312 | 0.682* |
| Lakeside Container 2017 | *A. alternata* | -0.063 | 0.586* | 0.610* | -0.406 | -0.225 | 0.434 |
|  | *Alternaria* spp. | 0.824* | -0.011 | 0.205 | 0.145 | 0.620* | 0.790* |
| Worcester 2017 | *A. alternata* | -0.144 | -0.467 | -0.388 | 0.268 | -0.033 | -0.327 |
|  | *Alternaria* spp*.* | 0.247 | 0.305 | 0.402 | -0.020 | 0.154 | 0.464 |
| Lakeside Container 2018 | *A. alternata* | 0.152 | 0.371 | 0.407 | -0.313 | -0.079 | 0.079 |
|  | *Alternaria* spp. | 0.687* | 0.869* | 0.808* | -0.625* | 0.097 | 0.648* |
| Worcester 2018 | *A. alternata* | -0.259 | 0.389 | 0.531 | -0.654* | -0.824* | -0.084 |
|  | *Alternaria* spp*.* | 0.748* | 0.784* | 0.731* | -0.488 | 0.067 | 0.673* |

*Significant correlations at *p*<0.05. OSR-Oilseed rape

**Table S3** Spearman’s correlation coefficients between weather parameters and weekly mean *A. alternata* and *Alternaria* spp. spore concentrations at the rural (Lakeside Container) and urban (Worcester) sites for the period 30 Jun-28 Sep 2016, 29 Jun-30 Sep 2017 and 12 Jul-19 Sep 2018

|  |  | **2016** |  |  |
| --- | --- | --- | --- | --- |
|  | **Lakeside container** | | **Worcester** | |
| **Parameters** | ***A. alternata*** | ***Alternaria* spp*.*** | ***A. alternata*** | ***Alternaria* spp.** |
| Wind direction (°) | -0.187 | -0.253 | -0.115 | -0.146 |
| Wind Speed (m/s) | -0.115 | -0.347 | -0.308 | -0.234 |
| Pressure (hPa) | 0.357 | 0.404 | 0.379 | 0.369 |
| Temperature (°C) | 0.456 | 0.660* | 0.549* | 0.660* |
| Dew Point (°C) | 0.077 | 0.418 | 0.242 | 0.349 |
| Precipitation (mm) | -0.212 | -0.183 | -0.314 | -0.377 |
| Relative humidity (%) | -0.621* | -0.300 | -0.451 | -0.495 |
| Solar radiation (W/m) | NA | NA | NA | NA |
| Leaf wetness | NA | NA | NA | NA |
|  |  | **2017** |  |  |
| Wind direction (°) | 0.624* | 0.323 | -0.345 | -0.309 |
| Wind Speed (m/s) | -0.098 | -0.159 | 0.091 | -0.455 |
| Pressure (hPa) | -0.133 | -0.093 | -0.109 | 0.582 |
| Temperature (°C) | 0.860* | 0.553* | 0.273 | 0.427 |
| Dew Point (°C) | 0.804* | 0.497* | 0.400 | 0.227 |
| Precipitation (mm) | -0.242 | 0.054 | 0.300 | -0.273 |
| Relative humidity (%) | -0.645* | -0.460* | 0.200 | -0.473 |
| Solar radiation (W/m) | 0.549* | 0.839* | NA | NA |
| Leaf wetness | -0.532* | -0.110 | NA | NA |
|  |  | **2018** |  |  |
| Wind direction (°) | 0.455 | -0.079 | -0.472 | -0.261 |
| Wind Speed (m/s) | -0.067 | -0.358 | 0.084 | -0.345 |
| Pressure (hPa) | -0.200 | -0.188 | -0.239 | -0.188 |
| Temperature (°C) | 0.697* | 0.903* | 0.420 | 0.818* |
| Dew Point (°C) | 0.782* | 0.745* | 0.032 | 0.709* |
| Precipitation (mm) | -0.188 | -0.188 | -0.110 | -0.030 |
| Relative humidity (%) | -0.442 | -0.673* | -0.653* | -0.515 |
| Solar radiation (W/m) | 0.418 | 0.867* | 0.213 | 0.721* |
| Leaf wetness | -0.806* | -0.467 | -0.006 | -0.406 |

*Significance level *p*<0.05. NA-No available data
